# Draft Genome Sequence of *Bifidobacterium lemurum* DSM 28807^T^ Isolated from the Gastrointestinal Tracts of Ring-Tailed Lemurs (*Lemur catta*)

**DOI:** 10.1128/genomeA.01656-16

**Published:** 2017-02-23

**Authors:** Hidehiro Toh, Takehiro Matsubara, Shuta Tomida, Iyo Mimura, Kensuke Arakawa, Takefumi Kikusui, Hidetoshi Morita

**Affiliations:** a Medical Institute of Bioregulation, Kyushu University, Fukuoka, Japan; b Okayama University Hospital Biobank, Okayama University Hospital, Okayama, Japan; c Graduate School of Medicine Dentistry and Pharmaceutical Sciences, Okayama University, Okayama, Japan; d Graduate School of Environmental and Life Science, Okayama University, Okayama, Japan; e School of Veterinary Medicine, Azabu University, Sagamihara, Japan

## Abstract

*Bifidobacterium lemurum* DSM 28807^T^ was isolated from the gastrointestinal tracts of ring-tailed lemurs (*Lemur catta*). Here, we report the first draft genome sequence of this organism.

## GENOME ANNOUNCEMENT

Bifidobacteria are Gram-positive bacteria with high G+C contents that are commonly found in the human and animal gastrointestinal tracts. Bifidobacteria are widely used as probiotic organisms, which confer a health benefit to the host when administered in adequate amounts. Genome sequences of bifidobacterial strains residing in the human gastrointestinal tract have been determined (1). However, studies of bifidobacteria of nonhuman primates are very few. Several studies recently have focused on bifidobacteria isolated from nonhuman primates. The novel species *Bifidobacterium lemurum* isolated from ring-tailed lemurs (*Lemur catta*) was reported (2). The 16S ribosomal RNA gene sequence of *B. lemurum* DSM 28807^T^, which was isolated from feces of a 5-year-old ring-tailed lemur (2), showed the highest similarity to that of *Bifidobacterium longum* subsp. *infantis* ATCC 15697^T^ (96%) and *Bifidobacterium breve* DSM 20213^T^ (96%). Thus, *B. lemurum* seems to belong to the *B. longum* group (3).

The *B. lemurum* DSM 28807^T^ genome was paired-end sequenced using Illumina’s MiSeq platform. Genomic libraries containing 600- to 1,000-bp inserts were constructed and sequenced, yielding 1,693,343 reads that provided 174-fold coverage of the genome. The sequence reads were assembled using CLC Genomics Workbench version 9.0.1, and the assembled genome consists of 47 contigs with a total length of 2,912,026 bp. This is the third largest bifidobacterial genome after *Bifidobacterium biavatii* (3.26 Mb) (4) and *Bifidobacterium scardovii* (3.16 Mb) (5) reported to date. The genome has a G+C content of 62.6%, which is the highest for a *Bifidobacterium* species. The draft genome of *B. lemurum* DSM 28807^T^ contained 2,322 predicted protein-coding genes. In the *B. longum* group, the genome of *B. lemurum* DSM 28807^T^ shared 1,445 and 1,400 protein-coding genes with those of *Bifidobacterium saguini* DSM 23967^T ^(4) and *Bifidobacterium reuteri* DSM 23975^T^ (4), respectively. In contrast, the genome of *B. lemurum* DSM 28807^T^ shared 1,309 and 1,299 protein-coding genes with those of *Bifidobacterium longum* subsp. *infantis* JCM 1222^T^ (6) and *Bifidobacterium breve* JCM 1192^T^ (7), respectively. Thus, *B. lemurum* DSM 28807^T^ shared more genes with the strains isolated from monkey gut (*B. saguini* and *B. reuteri*) than those from human gut (*B. longum* subsp. *infantis* and *B. breve*) in the *B. longum* group. *B. lemurum* DSM 28807^T^ contained more genes involved in carbohydrate transport and metabolism and transcription than bifidobacterial strains residing in the human gut. The genome information of this species will be useful for further studies of its physiology, taxonomy, and ecology.

### Accession number(s).

The draft genome sequence for *B. lemurum* DSM 28807^T^ has been deposited in the DDBJ/GenBank/EMBL database under accession numbers BDIS01000001 to BDIS01000047.
